# The Epidemiology of Alcoholic Liver Disease

**Published:** 2003

**Authors:** Robert E. Mann, Reginald G. Smart, Richard Govoni

**Affiliations:** Robert E. Mann, Ph.D., is a senior scientist in the Department of Social, Prevention and Health Policy Research at the Centre for Addiction and Mental Health and an associate professor in the Department of Public Health Sciences at the University of Toronto, both in Toronto, Canada. Reginald G. Smart, Ph.D., is a principal and senior scientist in the Department of Social, Prevention and Health Policy Research at the Centre for Addiction and Mental Health in Toronto, Canada. Richard Govoni, Ph.D., is a research fellow in the Department of Public Health Sciences at the University of Toronto and an assistant professor in the Department of Psychology at the University of Windsor in Windsor, Canada

**Keywords:** alcoholic liver cirrhosis, epidemiological indicators, gender differences, ethnic differences, AODR (alcohol and other drug related) mortality, morbidity, AOD (alcohol and other drug) use pattern, risk factors, trend, aggregate AOD consumption, beneficial vs adverse drug effect, Alcoholics Anonymous, United States, survey of research

## Abstract

This article describes the various forms of alcoholic liver disease (ALD), with particular emphasis on cirrhosis, the form of liver disease that often is most associated with alcohol abuse and about which the most information is available. Epidemiological research has evaluated the prevalence of ALD and the factors that often contribute to the disease. Although the most potent factor in ALD is the excessive consumption of alcoholic beverages, gender and ethnic differences also account for some important variations in rates of liver disease. Mortality rates from cirrhosis have declined in the United States and some other countries since the 1970s. A number of factors may have contributed to this decline, including increased participation in treatment for alcohol problems and Alcoholics Anonymous membership, decreases in alcohol consumption, and changes in the consumption of certain types of alcoholic beverages.

One of the most enduring insights into the effects of alcohol has been the assertion that heavy alcohol consumption increases mortality rates, especially those from cirrhosis of the liver and other forms of liver disease (see the sidebar, p. 211). The scientific study of alcohol-related mortality began in the 1920s with Pearl’s studies (1926) of death rates among various types of drinkers. He and others found that heavy drinkers had higher rates of overall mortality and of mortality from cirrhosis than did lighter drinkers or abstainers. Since then, mortality studies have continued to demonstrate that heavy drinkers and alcoholics die from cirrhosis at a much higher rate than the general population (Mann et al. 1993; Pell and D’Alonzo 1973; Schmidt and de Lint 1972; Thun et al. 1997). In addition, laboratory studies conducted in the 1930s established that feeding large amounts of alcohol to rats and other animals caused liver disease (Lelbach 1974).

**Types of Alcoholic Liver Disease**

The most prevalent types of alcoholic liver disease are fatty liver, alcoholic hepatitis, and cirrhosis. Often, as people continue to drink heavily, they progress from fatty liver to hepatitis to cirrhosis. The disorders can also occur together, however, and liver biopsies can show signs of all three in some people (Kirsh et al. 1995).

***Alcoholic Fatty Liver***

About 20 percent of alcoholics and heavy drinkers develop fatty liver, or steatosis. In many cases there are no clinical symptoms except for an enlarged liver (hepatomegaly). Fatty liver can be reversed if alcohol consumption is stopped or significantly reduced, but the condition can lead to death if alcohol consumption is not reduced or stopped. Some biopsies from people with fatty liver show inflammatory changes, an early sign of more serious liver disease.

***Alcoholic Hepatitis***

Alcoholic hepatitis usually is diagnosed when a liver biopsy indicates inflammatory changes, liver degeneration, fibrosis, and other changes to liver cells. Common clinical signs of alcoholic hepatitis include swollen liver, nausea, vomiting, and abdominal pain. Patients also may experience fever, jaundice, liver failure, and bleeding. The rate of mortality in severe cases is about 50 percent. If heavy drinking continues, about 40 percent of cases of alcoholic hepatitis will develop into cirrhosis.

***Alcoholic Cirrhosis***

Cirrhosis of the liver is the most serious form of ALD and a cause of many deaths and serious illnesses. In cirrhosis, scar tissue replaces normal liver tissue, disrupting blood flow through the liver and preventing it from working properly. Clinical signs of cirrhosis include redness of the palms caused by capillary dilation (palmar erythema); shortening of muscles in the fingers (contractures) caused by toxic effects or fibrous changes; white nails; thickening and widening of the fingers and nails (clubbing); liver enlargement or inflammation; and abnormal accumulation of fat in normal liver cells (fatty infiltration). Diagnosis of cirrhosis must be made with biopsies, although laboratory tests can be helpful as well.

About 10 percent to 15 percent of people with alcoholism develop cirrhosis, but many survive it. Many are unaware that they have it, and about 30 percent to 40 percent of cirrhosis cases are discovered at autopsy (Anand 1999). The 5-year survival rate for people with cirrhosis who stop drinking is about 90 percent, compared with 70 percent of those who do not stop drinking. However, for late-stage cirrhosis—that is, when jaundice, accumulation of fluid in the abdomen (ascites), or gastrointestinal bleeding have occurred—the survival rate is only 60 percent for those who stop drinking and 35 percent for those who do not.

***Other Forms of Liver Disease Affected by Alcohol***

Alcohol can be a factor in other forms of liver disease not specifically attributed to it, and alcohol may interact with risk factors for other forms of liver disease. For example, people with alcohol-related cirrhosis are at much higher risk for the development of liver cancer (Hall 1995). Likewise, heavy drinking in combination with hepatitis B or C substantially increases the risk of liver cirrhosis, compared with the risk associated with heavy drinking alone (Corrao et al. 1998).

— Robert E. Mann, Reginald G. Smart, and Richard Govoni

ReferencesAnandBSCirrhosis of the liverWestern Journal of Medicine171110115199910510657PMC1305772CorraoGZambonATorchioPAttributable risk for symptomatic liver cirrhosis in ItalyJournal of Hepatology286086141998956682910.1016/s0168-8278(98)80284-5HallPPathological spectrum of alcoholic liver diseaseHallPAlcoholic Liver Disease: Pathobiology and Pathogenesis2d edLondonEdward Arnold19954168KirshRRobsonSTreyCDiagnosis and Management of Liver DiseaseLondonChapman and Hall1995

Alcohol consumption increased substantially in many countries after World War II, which spurred greater interest in the effects of alcohol consumption on cirrhosis and other forms of alcoholic liver disease (ALD). One of the most influential efforts to summarize research in this area was undertaken in 1975 by an international group of scientists sponsored by the World Health Organization (WHO). The resulting book, *Alcohol Control Policies in Public Health Perspective* (Bruun et al. 1975), reviewed studies of clinical and nonclinical populations of heavy drinkers. All studies found that a greater proportion of heavy drinkers died of cirrhosis than would be expected based on rates of cirrhosis deaths in the general population (i.e., liver cirrhosis deaths among heavy drinkers ranged from 2 to 23 times higher than the rate that would be expected in the general population).

This research established a firm connection between heavy alcohol consumption and liver disease. Investigators also have observed that the price of alcohol is a significant determinant of alcohol consumption and thus of cirrhosis mortality rates (Bruun et al. 1975; Edwards et al. 1994; Seeley 1960). These findings have laid the foundation for an influential public health approach to controlling liver disease and other alcohol problems that emphasizes the control of alcohol’s availability and includes recommendations to control cirrhosis and other alcohol-related problems through taxation (Chaloupka et al. 2002; Cook and Tauchen 1982). The validity of this availability-control approach has been widely supported (e.g., Edwards et al. 1994), and investigations of the epidemiology of ALD have continued to be central to it (e.g., Ramstedt 2001).

**Ode to the Liver**

*Modest,*
*organized*
*friend,*
*underground*
*worker,*
*let me give you*
*the wing of my song,*
*the thrust*
*of the air,*
*the soaring*
*of my ode:*
*it is born*
*of your invisible*
*machinery,*
*it flies*
*from your tireless*
*confined mill,*
*delicate*
*powerful*
*entrail,*
*ever alive and dark.*
*While*
*the heart resounds and attracts*
*the music of the mandolin,*
*there, inside,*
*you filter*
*and apportion,*
*you separate*
*and divide,*
*you multiply*
*and lubricate,*
*you raise*
*and gather*
*the threads and the grams*
*of life, the final*
*distillate,*
*the intimate essences.*
*Submerged*
*viscus,*
*measurer*
*of the blood,*
*you live*
*full of hands*
*and full of eyes,*
*measuring and transferring*
*in your hidden*
*alchemical*
*chamber.*
*Yellow*
*is the matrix*
*of your red hydraulic flow,*
*diver*
*of the most perilous*
*depths of man,*
*there forever hidden,*
*everlasting,*
*in the factory,*
*noiseless.*
*And every feeling*
*or impulse*
*grew in your machinery,*
*received some drop*
*of your tireless*
*elaboration,*
*to love you added*
*fire or melancholy,*
*let one tiny cell*
*be in error*
*or one fiber be worn*
*in your labor*
*and the pilot flies into the wrong sky,*
*the tenor collapses in a wheeze,*
*the astronomer loses a planet.*
*Up above, how*
*the bewitching eyes of the rose*
*and the lips*
*of the matinal carnation*
*sparkle!*
*How the maiden*
*in the river laughs!*
*And down below,*
*the filter and the balance,*
*the delicate chemistry*
*of the liver,*
*the storehouse*
*of the subtle changes:*
*no one*
*sees or celebrates it,*
*but, when it ages*
*or its mortar wastes away,*
*the eyes of the rose are gone,*
*the teeth of the carnation wilted*
*and the maiden silent in the river.*
*Austere portion*
*or the whole*
*of myself,*
*grandfather*
*of the heart,*
*generator*
*of energy:*
*I sing to you*
*and I fear you*
*as though you were judge,*
*meter,*
*implacable indicator,*
*and if I can not*
*surrender myself in shackles to austerity,*
*if the surfeit of*
*delicacies,*
*or the hereditary wine of my country*
*dared*
*to disturb my health*
*or the equilibrium of my poetry,*
*from you,*
*dark monarch,*
*giver of syrups and of poisons,*
*regulator of salts,*
*from you I hope for justice:*
*I love life: Do not betray me! Work on!*
*Do not arrest my song.*

Pablo Neruda, 1904–1973

Nobel Laureate in Literature, 1971

Translation by Oriana Josseau Kalant

*“Oda al Higado,”* by Pablo Neruda, translated by Oriana Josseau Kalant, as published in *Alcohol Liver Pathology* (J.M. Khana, Y. Israel, and H. Kalant, editors) © 1975. Reprinted with permission of the Centre for Addiction and Mental Health, Toronto.

## Drinking Patterns and Alcoholic Liver Disease

Many studies show that the amount of alcohol consumed and the duration of that consumption are closely associated with cirrhosis.^1^ One of the best demonstrations of this association was presented by Lelbach (1974), who studied 319 patients in an alcoholism clinic in Germany. He calculated the average amount of alcohol consumed per hour in a 24-hour day. As shown in table 1, patients with normal liver function consumed far less alcohol than did those with cirrhosis. Those who did not have cirrhosis but did have other liver malfunctions had intermediate rates of alcohol intake. In addition, patients with normal liver function had been drinking heavily for only about 8 years on average, whereas those with cirrhosis had been drinking heavily for more than 17 years on average. As this research illustrates, the risk of developing cirrhosis is a function of both quantity and duration of alcohol consumption. Bellentani and Tiribelli (2001) recently proposed that cirrhosis does not develop below a lifetime alcohol ingestion of 100 kg of undiluted alcohol. This amount corresponds to an average daily intake of 30 grams of alcohol (between two and three drinks^2^) for 10 years. These investigators also noted that consuming alcohol with food resulted in somewhat lower levels of risk than consuming alcohol by itself.

**Figure 1:**
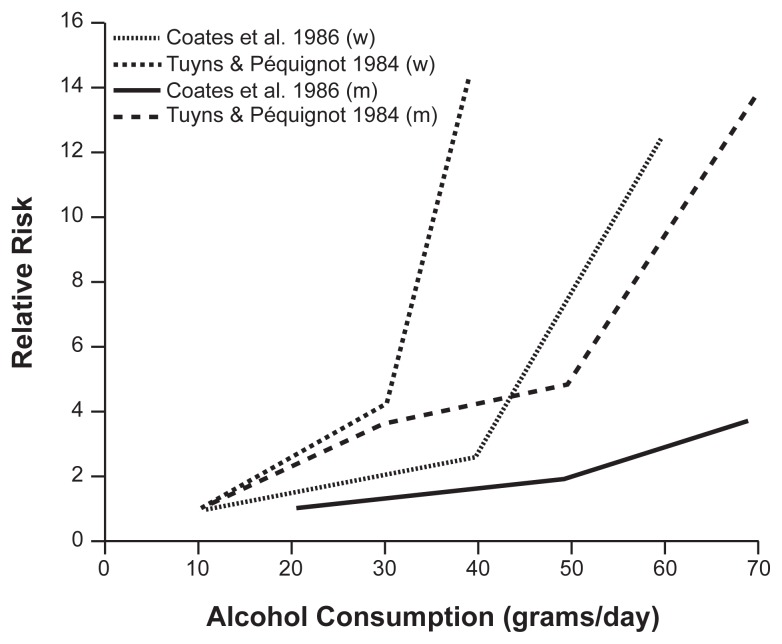
Alcohol consumption and incidence of cirrhosis of the liver in men (m) and women (w). Studies have shown a close relationship between alcohol consumption and cirrhosis risk. NOTE: Data truncated at 70 g/day.

More recent studies confirm the close association between alcohol consumption and cirrhosis risk. Anderson (1995) examined data from four case control studies in men and women. (Figure 1 shows results from representative studies [Coates et al. 1986, and Tuyns and Péquignot 1984].) This investigation showed that the risk of cirrhosis was related to the amount of alcohol consumption in every study. In addition, as alcohol consumption increased, the risk of cirrhosis increased more rapidly for females than it did for males. The link between gender and risk for cirrhosis is addressed in detail in the section on page 215.

## Cirrhosis Morbidity and Mortality and Average Alcohol Consumption

The strong link between heavy or excessive alcohol use and the development of liver disease took on added significance in the middle of the 20th century, when several researchers began exploring cirrhosis as a potential marker for levels of alcohol problems in populations (Jellinek and Keller 1952; Ledermann 1956; Seeley 1960; Terris 1967). Of particular importance was the discovery of a relationship between cirrhosis mortality rates and per capita levels of alcohol consumption in the population. This relationship has proved to be remarkably strong and has been consistently observed across time periods and in various regions of the world (Bruun et al. 1975; Ramstedt 2001; Smart and Mann 1991). European researchers have observed a lagged relationship between cirrhosis mortality and consumption measures, with the rate of cirrhosis mortality in a year being influenced by the alcohol consumption rates of several previous years (Corrao 1998; Ramstedt 2001). To account for this effect, Skog (1980) developed a “distributed lag model,” in which the effects of alcohol consumption in a year are distributed over the next several years. Using this model, he was able to explain an apparent inverse relationship between consumption and cirrhosis mortality rates in Great Britain between 1931 and 1958 (Popham 1970). Incorporating the distributed lag model into the data produced the expected positive relationship between consumption and cirrhosis mortality.

## Trends in Cirrhosis Mortality Rates

Liver cirrhosis is a major cause of death in the United States (Yoon et al. 2002; Minino et al. 2002). In 2000, it was the 12th leading cause of death, accounting for 1.1 percent of all deaths, with an age-adjusted death rate^3^ of 9.6 per 100,000 population. Cirrhosis mortality rates vary substantially among age groups: They are very low among the young but increase considerably in middle age, reaching a peak of 31.1 per 100,000 among people ages 75 to 84. Because of the increase in cirrhosis mortality rates in middle age, the contribution of cirrhosis to total deaths reaches a peak in the 45–54 age group, for which it is the fourth leading cause of death. In relation to the cirrhosis mortality rate in other countries, the United States is in the middle range, as are countries such as Belgium and Canada (WHO 2000). Higher rates are seen in countries where people traditionally consume more alcohol than in the United States, such as Spain, France, and Italy. In countries where alcohol consumption is traditionally lower—Iceland, New Zealand, and Norway, for example— cirrhosis death rates are lower.

**Figure 2:**
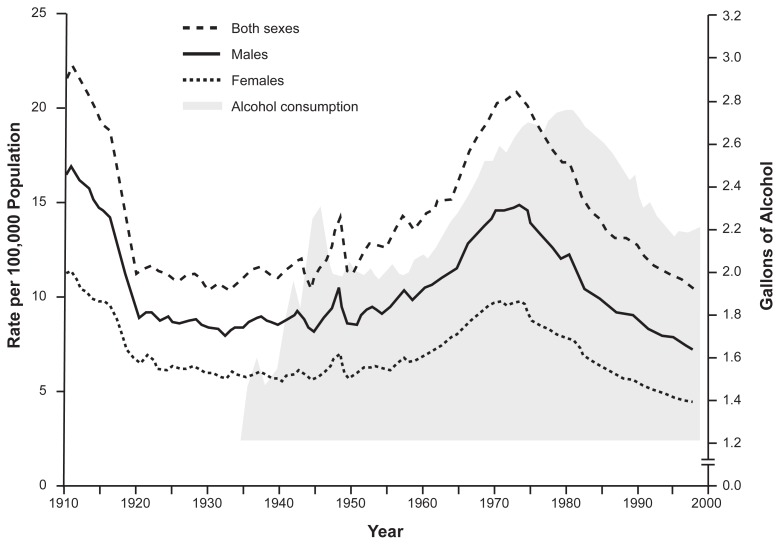
Age-adjusted death rates of liver cirrhosis by gender, 1910–1932 in death registration States, and 1933–1977 in entire United States. U.S. cirrhosis mortality rates were high at the beginning of the 20th century, declined precipitously with the introduction of Prohibition, and increased again when Prohibition ended. Mortality rates continued to increase until the early to mid-1970s, when these rates began to approach the levels seen in the first decade of the century. In the mid-1970s cirrhosis mortality rates began to decline again, as they had with the introduction of Prohibition, and they have continued to decline. INSET (shaded area): Per capita alcohol consumption for the years 1935 to 1999, illustrating the link between alcohol consumption and cirrhosis mortality. SOURCES: Mortality rate data adapted from Yoon et al. 2001; consumption data from Nephew et al. 2002.

Cirrhosis mortality rates in the United States have changed substantially over time. Early in the 20th century, these rates were at their highest point. As shown in figure 2, overall cirrhosis mortality rates declined precipitously with the introduction of Prohibition. When Prohibition ended, alcohol consumption and cirrhosis mortality rates increased until the late 1960s and early 1970s, when these rates began to approach levels seen in the first decade of the century. However, in the mid-1970s cirrhosis mortality rates began to decline as they had with the introduction of Prohibition; cirrhosis was the 8th leading cause of death in 1977 (Galambos 1985) but the 12th leading cause of death by 2000. Similar declines in cirrhosis mortality rates have been observed in many developed countries (including Canada, Sweden, France, and Italy), but in other developed countries (e.g., Great Britain, Finland, Denmark) cirrhosis death rates have increased (Ramstedt 2001). The reasons for the dramatic reductions remain a source of considerable interest, as will be discussed below.

Cirrhosis mortality rates may continue to decline if alcohol consumption rates remain low or fall further. However, the increase in cases of hepatitis C infection in the United States, which are predicted to peak by 2015 (Armstrong et al. 2000), may affect the rate of cirrhosis deaths. Because people infected with hepatitis C are more likely to develop cirrhosis when they drink, death rates from cirrhosis may increase in the future, even if drinking levels decline. (For more information on hepatitis C infection and alcohol, see the article by Schiff and Ozden in this issue.)

## Reasons for Decreases in Cirrhosis Death Rates

### Changes in Per Capita Alcohol Consumption

Changes in per capita consumption of alcohol must be considered a leading candidate for the cause of recent reductions in cirrhosis mortality rates. Research demonstrates that, over a long period, changes in per capita consumption are broadly consistent with changes in cirrhosis mortality rates (Ramstedt 2001; Singh and Hoyert 2000; Xie et al. 2000) (see figure 2). However, cirrhosis mortality in the United States, Canada, and some other regions began to decline in the mid-1970s, before per capita consumption rates began to go down (also see figure 2). This is the opposite of what would be expected based on the hypothesized lagged relationship between per capita consumption and cirrhosis mortality rates. Thus, researchers are considering whether other factors also have influenced cirrhosis mortality rates in recent years.

### Beverage-Specific Effects

The relationship between cirrhosis mortality and alcohol consumption may vary depending on the type of alcoholic beverage—beer, wine, or spirits— consumed. Any such beverage-specific effects could help explain why cirrhosis mortality began to decline in the 1970s despite the continued rise in total alcohol consumption. Researchers over the past four decades have investigated this question (e.g., Terris 1967; Gruenewald and Ponicki 1995; Schmidt and Bronetto 1962).

Recently, Roizen and colleagues (1999) and Kerr and colleagues (2000) have proposed that cirrhosis mortality is more strongly associated with consumption of spirits than with other alcoholic beverages, and that this relationship accounts for the apparent discrepancy between per capita alcohol consumption measures and cirrhosis mortality rates. Roizen and colleagues (1999) examined U.S. cirrhosis mortality data from 1949 to 1994 and observed that consumption of spirits was more strongly related to cirrhosis mortality than was total alcohol consumption, a finding that is consistent with earlier observations of U.S. data (Terris 1967). Kerr and colleagues (2000) extended this analysis to several other countries, with similar results. The relationship between spirits consumption and cirrhosis mortality during the 1970s, when cirrhosis mortality rates began to decline in the United States, suggests that the discrepancy between cirrhosis rates and per capita alcohol consumption observed at that time arose because research did not focus on spirits, the beverage most strongly related to cirrhosis mortality.

The stronger association between cirrhosis mortality and consumption of spirits may be attributable to biological and sociobehavioral mechanisms. Some types of alcoholic beverage may be more toxic to the liver than others (Lelbach 1974; Schmidt and Bronetto 1962). In addition, consumption of certain alcoholic beverages may be associated with different drinking styles (Smart 1996)—that is, people who tend to drink frequently and heavily, and thus are at greatest risk for developing cirrhosis, also may tend to drink spirits rather than beer or wine. Thus, drinking style may collude with biological mechanisms to significantly raise some drinkers’ risk of liver disease. This interesting and important issue is the subject of ongoing investigation.

### Increased Participation in Treatment and Alcoholics Anonymous Programs

Another possible reason for declines in cirrhosis mortality has been increased participation in treatment for alcohol abuse and in Alcoholics Anonymous meetings (Mann et al. 1988*a*, *b*; Holder and Parker 1992; Romelsjö 1987; Smart and Mann 2000). Specifically, cirrhosis morbidity and mortality rates could be influenced if participation in alcoholism treatment and AA are in some degree effective in reducing excessive drinking among heavy or abusive drinkers, if sufficient treatment occurs, and if enough alcoholics become members of AA or receive other treatment services. The 1970s and 1980s saw large increases in AA participation and in the number of people who received alcoholism treatment services (Mann et al. 1988*b*, 1991). Smart and Mann (1993) examined whether these increases in treatment and AA participation could affect cirrhosis morbidity and mortality rates. According to estimates derived from the research:

- Alcoholics seeking treatment drink an average of 160 g of undiluted alcohol per day.
- About 14 percent of alcoholics will develop cirrhosis if they drink this quantity for a period of 8 years.
- About 50 percent of alcoholics receiving treatment or attending AA meetings improve sufficiently to postpone the development of cirrhosis or avoid death if they already have cirrhosis.

The authors applied these figures to the actual number of people who were AA members or were receiving alcohol abuse treatment in 1975 and 1986 in Ontario and the United States. Based on this analysis, between 25 percent and 100 percent of the actual reduction in cirrhosis deaths and hospital discharges during the period could be predicted, depending on the degree of overlap between treatment and AA membership that was assumed.

Other studies of the relationship between cirrhosis mortality rates and aggregate, or population, levels of treatment and AA membership rates support the hypothesis that increases in treatment and AA membership helped reduce cirrhosis mortality rates, both in the United States and elsewhere. Several studies (for a review, see Smart and Mann 2000) have found an association between reductions in cirrhosis morbidity and mortality and increased levels of treatment and AA membership in Canada (Mann et al. 1988*b*), the United States (Mann et al. 1991), and Sweden (Leifman and Romelsjö 1997; Romelsjö 1987). Examining monthly cirrhosis mortality data from North Carolina, Holder and Parker (1992) found that alcohol abuse treatment had a significant short-term lagged relationship with cirrhosis mortality, with an increase in treatment being followed 3 months later by a decline in cirrhosis mortality. Finally, Smart and colleagues (1996) found that increased funding for alcoholism treatment was associated with cirrhosis mortality reductions across the United States. Thus, the data so far provide strong support for the proposition that if a large enough portion of the population participates, AA membership and alcohol abuse treatment can influence cirrhosis morbidity and mortality rates at the population level.

## Other Factors Associated With Increased Rates of Cirrhosis Morbidity and Mortality

### Gender Differences

As discussed above, historically the epidemiology of cirrhosis has been linked closely to types and patterns of alcohol consumption. Other factors also may be at work in the development of liver disease. For example, there are important and long-standing gender differences in cirrhosis mortality risk and mortality rates. As shown in figure 2, cirrhosis mortality rates are about two times higher in men than in women. These rates reflect the fact that men typically drink more than women, and that the proportion of heavy drinkers and alcoholics is much higher among men. However, as noted previously, it also appears that at any given level of alcohol consumption, women have a higher likelihood of developing cirrhosis than men (see figure 1) (Tuyns and Péquignot 1984). This phenomenon is poorly understood, but several possible explanations have been offered. One is that levels of alcohol dehydrogenase, an enzyme involved in breaking down alcohol, may be lower in the stomachs of females than in males, which would result in a higher blood alcohol content for females than for males who consume equivalent amounts of alcohol (Frezza et al. 1990). Because damage to the liver is a function of blood alcohol levels and exposure time, factors that lead to higher blood alcohol concentrations could at least partially explain females’ higher risk for alcohol-related cirrhosis. Another possible explanation is that estrogen may increase the susceptibility of the liver to alcohol-related damage (Ikejima et al. 1998; Colantoni et al. 2003). Behavioral factors, including drinking patterns and diet, also may contribute to females’ higher cirrhosis risk.

Genetic factors, including those that influence alcohol metabolism and risk for alcoholism, also may be involved in the increased risk for cirrhosis seen in women (Reed et al. 1996), but there still is considerable debate on this issue, and further research is needed on the nature and the extent of such genetic contributions.

In a recent study, Corrao and colleagues (1998) found that 98.1 percent of cirrhosis cases in men but only 67.0 percent of cases in women could be attributed to alcohol consumption, hepatitis C, and hepatitis B. The risk factors for cirrhosis appear to be more complex for women than they are for men, and more research will be required to identify and understand them.

### Ethnic Differences

**Figure 3:**
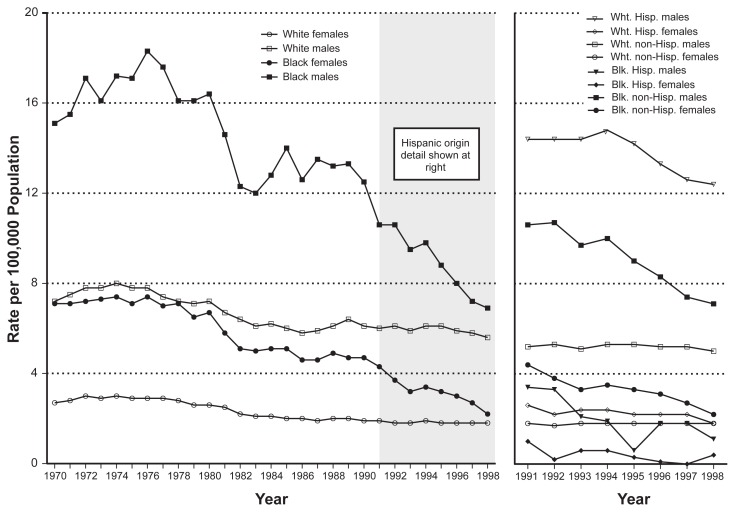
Age-adjusted rates of alcohol-related cirrhosis by gender and ethnic group (Black, White, and Hispanic), United States, 1970–1998. SOURCE: Yoon et al. 2001. (Categories shown in this figure were those used in the source study.)

Important differences in cirrhosis rates and cirrhosis mortality also exist among ethnic groups. Although ethnic group differences have been declining in recent years, cirrhosis rates remain higher for Blacks than for Whites in the United States (see figure 3), and the highest cirrhosis mortality rates currently are observed among Hispanic groups (Stinson et al. 2001). Although these differences in cirrhosis rates among Blacks, Whites, and Hispanics seem to suggest higher alcohol consumption levels among Hispanics and Blacks than among Whites, studies of alcohol consumption patterns in these groups tend not to support this interpretation. For example, in recent years, alcohol consumption among Blacks has been less than or comparable with that of Whites (see table 2).

Several reasons for ethnic group differences in cirrhosis rates have been proposed, including demographic factors related to gender, age, income, education, and employment; biological factors, such as family history of drinking problems; and environmental factors, such as stress (for a review, see Jones-Webb 1998). Other suggested factors are differential access to alcoholism treatment services (Singh and Hoyert 2000), although as yet no data are available to support this explanation; and differing rates of hepatitis C infection, which appears to be more prevalent among Hispanics than in Black and White populations (Yen et al. 2003). Ethnic group differences in cirrhosis risk and mortality may be linked to the possibility that, over time, general health status has improved more for some ethnic groups than others. However, as summarized in table 3, two general health indicators— age-adjusted death rate and life expectancy at birth—showed comparable gains for Blacks and Whites between 1970 and 2000. Thus, it is not yet possible to attribute changes in cirrhosis rates to changes in general health indicators of various groups.

As this discussion indicates, cirrhosis rates in subpopulations, such as those based on gender or ethnicity, can show significant deviations from the rates of cirrhosis that would be expected from alcohol consumption levels alone. These differences, which are not yet well understood, have important implications for research and prevention initiatives. From a public health perspective, an understanding of subpopulation dynamics is critical to the development of programs for preventing alcoholic liver disease.

## Conclusion

Alcoholic liver disease is a major source of alcohol-related morbidity and mortality. Heavy drinkers and alcoholics may progress from fatty liver to alcoholic hepatitis to cirrhosis, and it is estimated that 10 percent to 15 percent of alcoholics will develop cirrhosis. The likelihood of developing ALD is, to a large extent, a function of both the duration and amount of heavy drinking, and the per capita consumption of alcohol within populations has been shown to be a strong determinant of cirrhosis mortality rates. Recent studies also suggest that alcohol and hepatitis C may exert a multiplicative effect on risk for cirrhosis and other liver disease.

Although ALD remains a major cause of death, important declines in ALD death rates have been observed in recent years. Undoubtedly these declines were caused in part by changes in alcohol consumption rates, but because the mortality rate decline began when consumption was still increasing, other factors appear to be involved as well. To date, the evidence indicates that increases in participation in AA and other treatment for alcohol abuse have played an important role in reducing cirrhosis mortality rates. Other research has suggested that cirrhosis mortality rates may be more closely related to consumption of certain alcoholic beverages—specifically spirits—than to total alcohol consumption, and that beverage-specific effects can account for the fact that cirrhosis rates appeared to decrease although consumption rates were increasing in the 1970s. Important differences in ALD rates in men and women and among different ethnic groups have been found as well. Further research into these differences is likely to lead to improved prevention and treatment of alcohol-related liver disease.

## Figures and Tables

**Table 1:** Liver Function and Alcohol Intake

Liver Function	No. of Cases	Mean Daily Alcohol Intake (milligrams of alcohol/kilograms of body weight) per Hour	Average Duration of Alcohol Abuse (years)
Normal liver function	70	90	7.7
Uncomplicated fatty liver	118	109	7.8
Severe steatofibrosis with inflammatory reactions	48	127	10.3
Chronic alcoholic hepatitis	78	125	11.9
Cirrhosis	39	147	17.1

NOTES: Patients with normal liver function consumed far less alcohol and had been drinking for fewer years than those with cirrhosis. Those who did not have cirrhosis but did have other liver malfunctions had intermediate rates of alcohol intake. See sidebar, p. 211, for definitions of alcoholic liver disease.

SOURCE: Adapted from Lelbach 1974.

**Table 2:** Consumption Patterns for Blacks and Whites, 1984 and 1992

Consumption Level	1984		1992	
	
	Blacks (%)	Whites (%)	Blacks (%)	Whites (%)
**Males**				
Abstainer	29	23	35	28
Infrequent	13	13	6	9
Less frequent	12	16	19	21
Frequent	30	27	25	29
Frequent heavy	16	19	15	12
**Females**				
Abstainer	46	31	51	36
Infrequent	18	23	24	22
Less frequent	19	19	12	24
Frequent	13	23	8	15
Frequent heavy	4	4	5	3

NOTES: In recent years, alcohol consumption among Blacks has been comparable to or less than that of Whites.

Some columns do not total 100 percent because of rounding.

SOURCE: Adapted from Jones-Webb 1998.

**Table 3:** General Health Indicators for U.S. Blacks and Whites, 1970 and 2000

	Black Males	White Males	Black Females	White Females
Age-Adjusted Death Rate per 100,000 Population*				
1970	1,873.9	1,513.7	1,228.7	1,193.3
2000	1,377.8	1,018.2	947.9	739.1
Percent change	−26.5	−32.7	−22.9	−30.1
**Life Expectancy (years)**				
1970	60.0	68.0	68.3	75.6
2000	68.2	74.8	74.9	80.0
Percent change	+13.7	+10.0	+9.7	+5.8

* Standardized to 2000 age distribution.

NOTE: Between 1970 and 2000, Blacks and Whites showed comparable gains in age-adjusted death rate and life expectancy at birth.

SOURCE: Minino et al. 2002.

## Footnotes

1 In examining trends in alcoholic liver disease, some authors have considered only those cases directly attributable to alcohol (e.g., Douds et al. 2003). Other authors have determined that many cirrhosis deaths coded as not involving alcohol are in fact alcohol related (particularly for some age groups, including the middle-aged); thus these authors have examined total cirrhosis deaths when evaluating trends (e.g., Ramstedt 2001).

2 The National Institute on Alcohol Abuse and Alcoholism (NIAAA) defines a standard drink as 11–14 grams (g) of alcohol, which corresponds to approximately one shot of 80-proof alcohol (about 14 g alcohol), one glass of wine (11 g), or one 12-oz beer (12.8 g).

3 Age adjustment is a statistical method of adjusting for age differences, between populations or over time, that might otherwise distort mortality trends. In the case of chronic diseases, including cirrhosis of the liver, unadjusted mortality rates may appear to be higher for older populations than for younger populations because mortality rates are higher, on average, in older people.

## References

[b56-209-219] Anand BS (1999). Cirrhosis of the liver. Western Journal of Medicine.

[b57-209-219] Corrao G, Zambon A, Torchio P (1998). Attributable risk for symptomatic liver cirrhosis in Italy. Journal of Hepatology.

[b58-209-219] Hall P, Hall P (1995). Pathological spectrum of alcoholic liver disease. Alcoholic Liver Disease: Pathobiology and Pathogenesis.

[b59-209-219] Kirsh R, Robson S, Trey C (1995). Diagnosis and Management of Liver Disease.

